# Topical and subconjunctival anesthesia versus topical anesthesia alone in patients with senile cataracts undergoing phacoemulsification: a double-blind randomized controlled trial

**DOI:** 10.1186/s12886-024-03284-1

**Published:** 2024-01-12

**Authors:** Wisaruta Wutthayakorn, Sunee Chansangpetch, Suppadech Tunruttanakul

**Affiliations:** 1 Department of Ophthalmology, Sawanpracharak Hospital, Nakhon Sawan, Thailand; 2 Center of Excellent in Glaucoma, Faculty of Medicine, Chulalongkorn University and King Chulalongkorn Memorial Hospital, Thai Red Cross Society, Bangkok, Thailand; 3 Department of Surgery, Sawanpracharak Hospital, 43 Atthakawee Road, Muang, Nakhon Sawan, 60000 Thailand

**Keywords:** Cataract, Local anesthesia, Phacoemulsification, Randomized controlled trial

## Abstract

**Background:**

This study compared topical anesthesia to a combination of topical anesthesia and subconjunctival anesthesia for phacoemulsification.

**Methods:**

This double-blinded parallel placebo-controlled randomized trial involved senile cataract patients scheduled for phacoemulsification between May and December 2022. Patients were randomly assigned to receive either topical anesthesia with 0.5% tetracaine hydrochloride and subconjunctival balanced salt solution injection (Control group) or topical anesthesia and subconjunctival injection with 2% lidocaine (Lidocaine group). Baseline parameters, cataract grades, and various outcomes were recorded, including pain scores at specific time points, patient cooperation scores, requests for additional anesthesia, and complications. Statistical methods included Fisher’s exact test, the *t*-test, ordinal logistic regression, and linear regression with robust standard errors.

**Results:**

In total, 176 patients were included in the study after excluding 33 patients. A significant reduction in immediate postoperative pain was achieved in the Lidocaine group (*p* < 0.001) and was maintained for 2 h (*p* = 0.011). Additionally, better cooperation was observed in this group (*p* = 0.038). However, patients in the Lidocaine group experienced more pain during the subconjunctival injection (*p* = 0.001) and a significant increase in subconjunctival hemorrhage related to the injection (*p* < 0.001). Despite this, the rates of surgical complications were comparable between the groups, and all phacoemulsification procedures were successfully completed using the assigned anesthetic technique.

**Conclusions:**

The addition of subconjunctival lidocaine injection to topical anesthesia reduced postoperative pain and improved patient cooperation during phacoemulsification. However, the lidocaine injection was painful, and it carried a higher risk of spontaneous-relief subconjunctival hemorrhage.

**Trial registration:**

Trial Registration Number: TCTR20220804003, date of registration August 4, 2022, retrospectively registered.

## Background

Cataracts represent a common ophthalmologic condition characterized by opacification of the lens of the eye. This opacity is typically caused by the accumulation of protein in the lens because of the aging process. Cataracts can result in visual impairment or blindness if left untreated [1].

Phacoemulsification is a commonly performed ophthalmic surgery for cataract removal that utilizes ultrasound energy to emulsify and extract the cataractous lens [2]. Various anesthesia modalities are employed to alleviate pain during the procedure ranging from topical anesthesia to regional anesthesia (or even general anesthesia in some atypical patients [e.g., mental disability, dementia]) [3]. Regional anesthesia techniques, such as retrobulbar and peribulbar anesthesia injection, induce transient akinesia (temporary loss of eye movement), facilitating the surgical process [4]. However, previous studies indicated that regional anesthesia might offer better pain control while carrying a higher risk of serious complications, including globe or optic nerve trauma and respiratory arrest [5]. Alternatively, local anesthesia serves as an acceptable alternative with a lower risk of anesthesia-related complications [6, 7].

Local anesthesia for phacoemulsification procedures can vary in complexity, and the procedures range from simple techniques such as topical anesthesia with or without subconjunctival anesthesia injection to more intricate methods such as intracameral or sub-tenon anesthesia [8]. Intracameral or sub-tenon anesthesia provides advantages such as transient akinesia (sub-tenon) and better pain control with comparable complications as topical anesthesia with or without subconjunctival anesthesia injection [8]. However, these two methods require specific anesthetic agents or instruments [9, 10]. Because of these considerations, local anesthesia with topical anesthesia and subconjunctival anesthesia injection is widely utilized as the primary anesthetic modality, at least in our country [11]. Despite the existence of published studies comparing different anesthesia modalities [12–14], to our knowledge, no study has directly compared topical anesthesia alone to topical anesthesia with subconjunctival anesthesia injection. Therefore, the objectives of this study were to assess and compare the efficacy of pain relief between these two approaches. Additionally, we simultaneously examined other relevant outcomes, including patient cooperation, complications, and the success of phacoemulsification procedures.

## Methods

### Design and setting

The study design was based on a double-blinded parallel placebo-controlled randomized trial. Participants treated from May to December 2022 were eligible. The setting was Sawanpracharak Hospital, a 700-bed tertiary hospital located in Nakhon Sawan, Thailand.

The study protocol was approved by the Sawanpracharak Hospital Ethical Committee for Research in Human Subjects (COA.14/2022). The study adhered to the tenets of the Declaration of Helsinki. Written informed consent was obtained from all patients before study enrollment. The study was registered with thaiclinicaltrials.org as TCTR20220804003. The first patient was enrolled once the study protocol received approval from the Sawanpracharak Hospital Ethical Committee for Research in Human Subjects. However, trial registration was performed after the enrollment process. This delay occurred because the study initiators were not aware of the policy set forth by the International Committee of Medical Journal Editors, which mandates prospective registration for all interventional clinical trials.

### Participants

All patients at least 40 years old who were diagnosed with senile cataracts were eligible. The exclusion criteria were as follows: pseudoexfoliation syndrome, lens subluxation, posterior polar cataract, poor pupil dilation, posterior synechiae, communication difficulties, dementia, mental retardation, deafness, medical conditions or postural deformities that prevent appropriate head positioning during surgery, neurological problems that impede the surgery (e.g., nystagmus, Parkinson’s disease), a lack of cooperation during the slit lamp examination, patients who were planned for extracapsular or intracapsular cataract extraction, or a concurrent need for any additional surgery (e.g., pterygium excision, trabeculectomy, goniosynechialysis, lysis posterior synechiae), allergy to topical or subconjunctival anesthesia, and refusal to participate in the study.

### Interventions

The phacoemulsification procedures were performed by a single surgeon (WW), an ophthalmologist and glaucoma specialist with more than 5 years of experience in this field. All eligible participants underwent a preoperative evaluation based on the study department’s protocol.

Participants were randomly assigned to the Control or Lidocaine group. Patients in both groups received topical anesthesia with 0.5% tetracaine hydrochloride drops. However, those in the Lidocaine group also received a subconjunctival injection of 0.2 ml of 2% lidocaine with adrenaline, whereas those in the Control group received 0.2 ml of balanced salt solution. Before entering the operating room, participants received at least three drops of a combined solution of topical 0.8% tropicamide and 5% phenylephrine hydrochloride, administered at 15-minute intervals. If the participant’s pupil was not fully dilated, an additional drop of topical 0.8% tropicamide and 5% phenylephrine hydrochloride was applied. Pupil dilation was conducted by an ophthalmic nurse before participants entered the operating room. Participants with insufficient pupil dilation were excluded from the study, as outlined in the exclusion criteria.

Upon entering the operating room, the anesthesia and preparation process began with the application of two drops of 0.5% topical tetracaine hydrochloride (administered 10 min apart) to the eye undergoing surgery. This was followed by a 0.2-ml subconjunctival injection at the superior conjunctiva of lidocaine with adrenaline in the Lidocaine group and balanced salt solution in the Control group. One additional drop of 0.5% tetracaine hydrochloride was then applied to both eyes before surgical site preparation and draping. The eye retractor was then inserted, and an additional drop of tetracaine hydrochloride was administered to the eye undergoing surgery. Additional topical tetracaine hydrochloride was available upon the patient’s request. Topical anesthesia was administered by an assistant nurse, whereas subconjunctival injection was performed by a surgeon.

The phacoemulsification technique used in this study involved temporal clear corneal phacoemulsification in all patients using the Infiniti® machine. Trypan blue was used for lens capsule dyeing in patients with mature cataracts or dense posterior subcapsular cataracts to facilitate capsulorhexis (surgical removal of the central anterior part of the eye’s lens capsule). Phacoemulsification of the lens nucleus, irrigation and aspiration of the cortex, and intraocular lens insertion were performed accordingly. For non-complicated cases, a single-piece intraocular lens was inserted into the capsular bag, whereas a three-piece intraocular lens was inserted in the sulcus for patients with posterior capsular rupture. Wound leaks, either at the main wound or a side port, were sutured with nylon 10 − 0 (additional corneal suturing).

The study interventions were performed as outpatient or ambulatory surgery.

### Variables and outcomes

Baseline parameters, such as sex, age, comorbidities, ocular underlying disease (glaucoma, non-proliferative diabetic retinopathy, and optic neuropathy), and cataract grading using the WHO simplified grading system (nuclear cataract [NUC] 1–3 and mature cataract) [15], were recorded. Only data from the first eye were analyzed if both eyes required phacoemulsification.

Other surgery-related parameters included whether the current surgery was cataract surgery for the second eye, the need for unplanned additional surgery, the requirement for corneal suturing, and the operative time. Unplanned additional surgery is an adjunct procedure performed to address intraoperative complications, such as anterior vitrectomy for posterior capsular rupture or the use of capsular hooks for intraoperatively detected lens subluxation caused by a weak lens zonule.

The primary outcome was the immediate postoperative pain score. Other pain-related outcomes were the pain score after topical anesthesia, pain score after subconjunctival injection, and pain score 2 h after surgery. Additional outcomes were the patient cooperation score and the need for additional topical anesthesia. Complications from subconjunctival injection and surgery were also documented. Pain was scored using a numeric rating scale (NRS, 0–10), with 0 indicating no pain and 10 indicating the most severe pain.

The immediate postoperative pain score was obtained at the end of the phacoemulsification procedure, whereas the pain score 2 h after surgery was obtained 2 h after the completion of surgery. The pain score after topical anesthesia was obtained after the second 0.5% topical tetracaine hydrochloride application but before the subconjunctival injection. The pain score after subconjunctival injection was obtained. The pain score when patients requested additional topical anesthesia was obtained when patients asked for additional topical tetracaine hydrochloride during the procedure.

The patient cooperation score was adapted from a prior study [16], and it ranged from 0 to 5. A score of 5 indicated that the patient’s cooperation was so poor that the surgeon could not continue with the surgery and general anesthesia had to be instituted, whereas a score of 0 denoted the best cooperation.

The pain score 2 h after surgery was assessed by a blinded assistant, whereas the participating surgeon evaluated all other pain scores, including the patient cooperation score.

### Sample size

Because no previous study results were available, a pilot study was conducted on 40 patients (20 in each group) to determine the expected pain scores after phacoemulsification surgery under either topical or subconjunctival anesthesia. The pilot study had the same inclusion and exclusion criteria with this study. In the study hospital, two groups of ophthalmologists routinely performed phacoemulsification using one of the two anesthesia methods. Patients were asked to report their immediate postoperative pain score. The pilot group receiving topical anesthesia had a mean pain score of 2.49 ± 1.92, whereas the pilot group receiving subconjunctival anesthesia had a mean pain score of 1.78 ± 1.39. Based on a significance level (α) of 0.05 and a power of 0.80, the required sample size for each group was calculated to be 88 patients.

### Randomization and blinding

A double-blinded, parallel, randomized trial with two balanced placebo-controlled groups was conducted in this study. The primary intervention being tested was the anesthesia technique, with both groups receiving topical anesthesia. The intervention (Lidocaine) group received an additional subconjunctival anesthesia injection, whereas the Control group received a sham injection of balanced salt solution. The randomization procedure involved computer-generated, permuted block randomization with block sizes of two. Each assignment was sequentially numbered and placed in a sealed opaque envelope. ST was responsible for generating the allocation sequence and sealing opaque envelopes. WW (the study main surgeon) enrolled the participants. A nurse assistant who was not involved in the study handled the envelope opening and prepared injection solutions according to the assigned results. The solutions were prepared using a 3-ml syringe with a 27-gauge needle containing 0.5 ml of balanced salt solution (Control group) or 2% lidocaine with adrenaline (Lidocaine group). Patients, the surgeon, and the assessor were all blinded to the randomization process and preparation of the injection solutions. Eye appearance was consistent between both study groups during outcome assessments since all participants underwent pupil dilation. Poor pupil dilation was a criterion for exclusion in our study.

### Statistical analysis

The data are presented as percentages, means ± standard deviations, or medians (interquartile ranges). Fisher’s exact test was used to compare categorical variables, and the *t*-test was used to compare continuous variables. Regarding statistical analysis of the main outcomes, considering the expected positive skewness of both the pain scores and the patient cooperation score, all pain scores were categorized regarding the correspondence of verbal descriptors and the NRS for pain intensity [17] as follows: mild pain, NRS = 1–4; moderate pain, NRS = 5–7; and severe pain, NRS = 8–10. The categorized NRS (two ordinal categorical outcome) was compared using ordinal logistic regression [18]. However, because of the absence of standardized patient cooperation scoring and categorization, the patient cooperation score was compared via linear regression with robust standard errors to calculate the effect size [19].

Post-hoc subgroup analyses were conducted to investigate whether three potential factors—cataract grade [20], second-eye operation [21], and prolonged surgery [22]—affected the pain scores immediately after surgery and patients’ cooperation. The cataract severity grade was divided into two groups: NUC1–3 and mature cataract groups. Due to the absence of specific criteria for prolonged phacoemulsification, we defined a prolonged procedure as one exceeding the 75th percentile of the entire population’s operative time. The same planned statistical analysis was used to compare the two groups. *p* < 0.05 indicated statistical significance. All statistical analyses were performed using Stata statistical software (StataCorp, College Station, TX, USA).

## Results

**Fig. 1:**
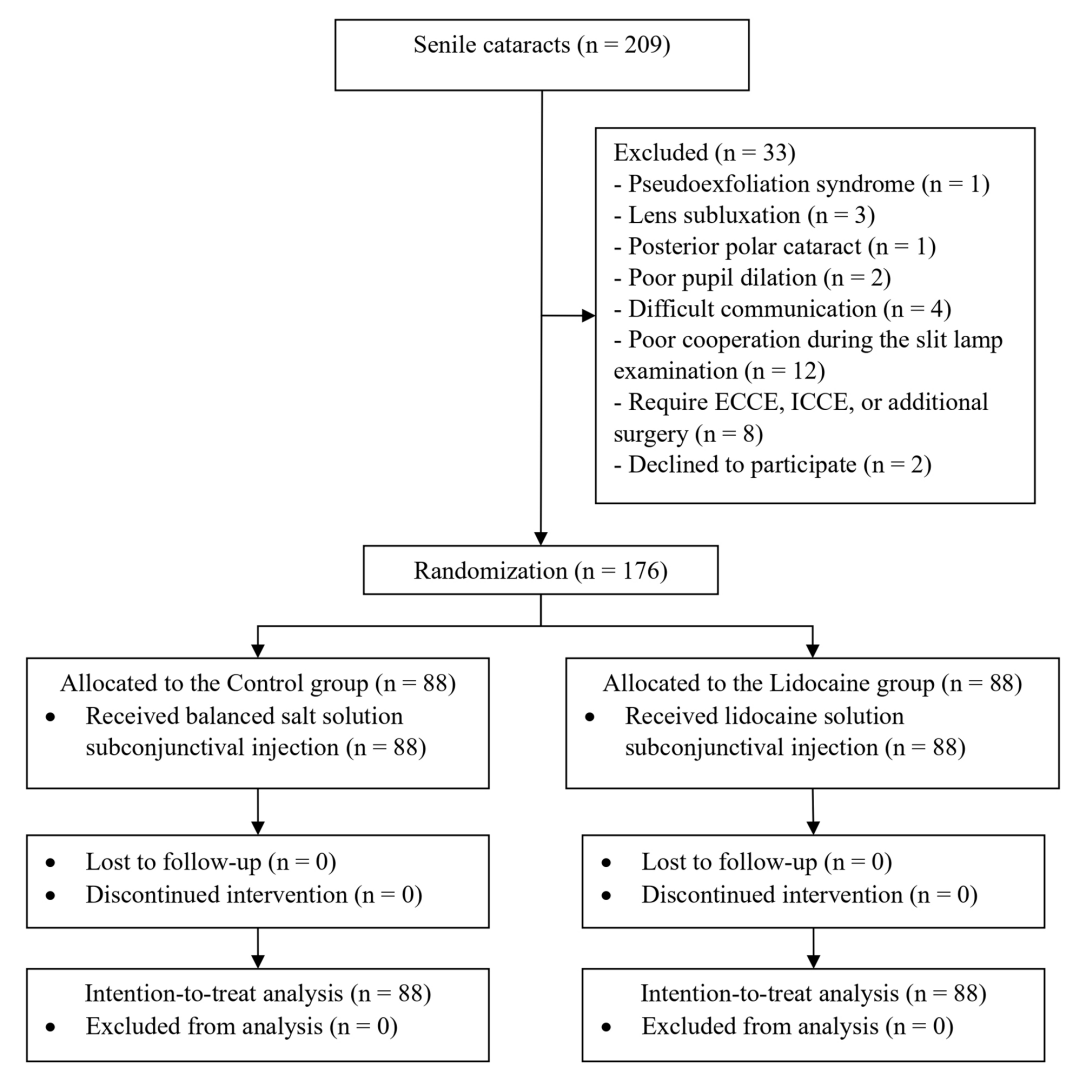
Flowchart of study participation. ECCE, extracapsular cataract extraction; ICCE, intracapsular cataract extraction

The flow of participants into the study is depicted in Fig. 1. Initially, 209 patients with senile cataracts were recruited during the study period. However, 33 patients who met the exclusion criteria were subsequently excluded. The remaining 176 patients were randomized to the Control or Lidocaine group (88 patients/group). There was no cross-intervention between the groups. All patients in both groups received complete follow-up, and there were no discontinued interventions. The study data collection and access for research purposes began in mid-May 2022 and ended in December 2022.

Table 1 provides a detailed overview of the baseline characteristics of the participants, and no imbalance was noted between the study groups. The phacoemulsification procedures were successfully accomplished in both groups in this study.

**Table 1:** The participants’ baseline characteristics and complications

	Control group (*n* = 88)	Lidocaine group (*n* = 88)	*p*
Sex, n (%)			
Male	39 (44.3)	37 (42.1)	0.879
Age (years), mean (SD)	66.6 (7.6)	65.8 (8.9)	0.501
Systemic underlying disease, n (%)			
Hypertension	41 (46.6)	48 (54.6)	0.366
Dyslipidemia	35 (39.8)	29 (33.0)	0.433
Diabetes mellitus	23 (26.1)	21 (23.9)	0.862
Chronic kidney disease	6 (6.8)	8 (9.1)	0.782
Underlying ocular disease, n (%)			
Glaucoma	9 (10.2)	10 (11.4)	> 0.999
NPDR	0 (0)	2 (2.3)	0.497
Optic neuropathy	1 (1.1)	0 (0)	> 0.999
Cataract grade, n (%)			0.267
NUC1	28 (31.8)	19 (21.6)	
NUC2	38 (43.2)	36 (40.9)	
NUC3	4 (4.6)	6 (6.8)	
Mature cataract	18 (20.5)	27 (30.7)	
Second eye operation*, n (%)	26 (29.6)	26 (29.6)	> 0.999
Unplanned additional surgery^†^, n (%)	1 (1.1)	2 (2.3)	0.497
Corneal suturing^‡^, n (%)	15 (17.1)	11 (12.5)	0.525
Operative time (min), mean (SD)	17.8(4.3)	17.8(5.7)	0.905
Complications, n (%)			
Complications related to the anesthetic technique: Subconjunctival hemorrhage	6 (6.8)	27 (30.7)	< 0.001
Surgical complications			0.371
- Posterior capsular rupture	0 (0)	2 (2.3)	
- Iris trauma	1 (1.1)	0 (0)	
- Subconjunctival hemorrhage	0 (0)	1 (1.1)	

^*^ Second eye operation: The current surgery was the second eye cataract operation

^†^ Unplanned additional surgery: An adjunct procedure was performed to address intraoperative complications (e.g., anterior vitrectomy or the use of capsular hooks)

^‡^ Corneal suturing: The need for corneal suturing secondary to wound leaks

NPDR, non-proliferative diabetic retinopathy; NUC, nuclear cataract; SD, standard deviation

**Fig. 2:**
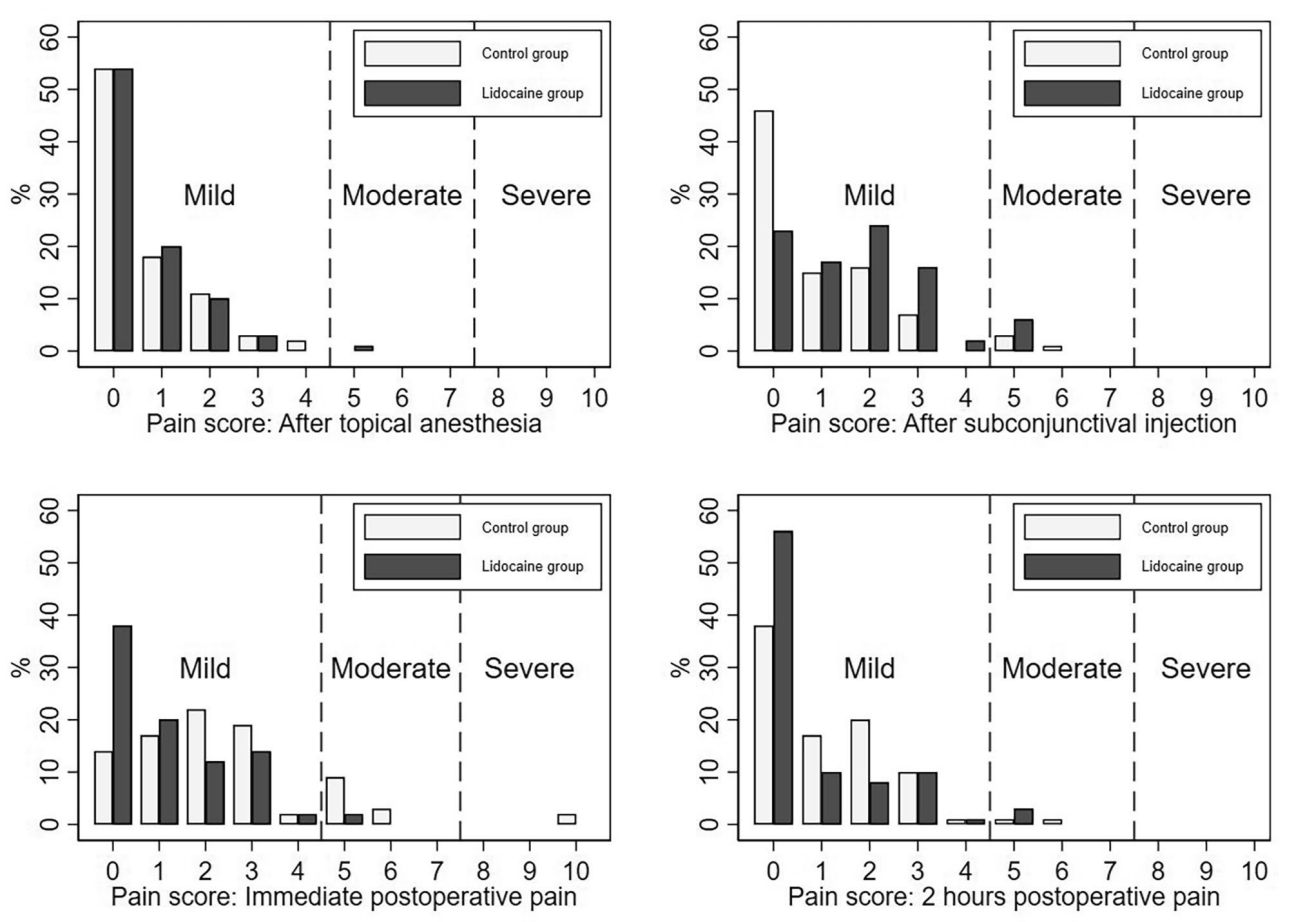
Pain scores during at various times with the corresponding severity grade based on verbal descriptors. Mild pain, pain score = 1–4; moderate pain, pain score = 5–7; severe pain, pain score = 8–10

Table 2 presents the main pain score results of the study, and the score distribution is illustrated in Fig. 2. The pain score immediately after surgery was significantly lower in the Lidocaine group than in the Control group. The percentages of patients with mild, moderate, and severe pain in the Control group were 68.2%, 13.6%, and 2.3%, respectively, whereas those in the Lidocaine group were 54.5%, 2.3%, and 0%, respectively (all *p* < 0.001). Notably, two patients in the Control group reported a maximum pain score of 10 immediately after surgery. The advantage of pain relief also persisted for approximately 2 h after surgery (*p* = 0.011). The Lidocaine group featured a higher proportion of patients experiencing no pain than the Control group (63.6% vs. 43.2%).

**Table 2:** Comparison of pain severity and cooperation scores between the study groups

Pain scores	Control group (*n* = 88) n (%)	Lidocaine group (*n* = 88) n (%)	*p*
After topical anesthesia			0.952^†^
- No pain	54 (61.4)	54 (61.4)	
- Mild	34 (38.6)	33 (37.5)	
- Moderate	0 (0)	1 (1.1)	
- Severe	0 (0)	0 (0)	
After subconjunctival injection			0.001^†^
- No pain	46 (52.3)	23 (26.1)	
- Mild	38 (43.2)	59 (67.1)	
- Moderate	4 (4.5)	6 (6.8)	
- Severe	0 (0)	0 (0)	
When patients requested additional topical anesthesia			0.132^†^
- No drug requested	83 (94.3)	87 (98.9)	
- Mild	2 (2.3)	1 (1.1)	
- Moderate	2 (2.3)	0 (0)	
- Severe	1 (1.1)	0 (0)	
Immediately after surgery			< 0.001^†^
- No pain	14 (15.9)	38 (43.2)	
- Mild	60 (68.2)	48 (54.5)	
- Moderate	12 (13.6)	2 (2.3)	
- Severe	2 (2.3)	0 (0)	
Two hours after surgery			0.011^†^
- No pain	38 (43.2)	56 (63.6)	
- Mild	48 (54.5)	29 (33.0)	
- Moderate	2 (2.3)	3 (3.4)	
- Severe	0 (0)	0 (0)	
Cooperation score^*^ (Median [IQR])	0 (0,1)	0 (0,1)	0.038^‡^
- Score difference (95% confidence interval)		−0.25 (− 0.49, − 0.01)	

^*^ The patient cooperation score ranged 0–5. A score of 5 indicated the worst cooperation, whereas a score of 0 denoted the best cooperation

^†^ Result was based on ordinal logistic regression

^‡^ Result was based on linear regression with robust standard errors

IQR, interquartile range

Although patients in the Lidocaine group experienced less postoperative pain overall, they reported more pain after the subconjunctival injection than those in the Control group, who received a balanced salt solution injection (*p* = 0.001). The Lidocaine group had larger proportions of patients with mild and moderate pain (67.1% and 6.8%, respectively), with only 26.1% of patients reporting no pain, whereas 52.3% of patients in the Control group reported no pain. Meanwhile, 43.2% and 4.5% of Control patients experienced mild and moderate pain, respectively.

Cooperation was significantly better in the Lidocaine group than in the Control group (mean score difference [95% confidence interval] = − 0.25 [− 0.49, − 0.01], *p* = 0.038).

Five patients (5.7%) in the Control group and one patient (1.1%) in the Lidocaine group required supplemental topical anesthesia during surgery (*p* = 0.210). One patient (1.1%) in the Control group needed two additional doses of anesthesia. The pain score among patients who requested additional topical anesthesia did not differ between the groups (*p* = 0.132).

Table 1 also provides an overview of surgical and anesthetic-related complications observed in both groups. The Lidocaine group had a significantly higher incidence of subconjunctival hemorrhage (anesthesia-related complication) than the Control group (30.7% vs. 6.8%, *p* < 0.001). The rates of surgical complications were similar between the groups and limited to patients with hard cataracts (*p* = 0.371). Specifically, posterior capsular rupture and surgical-related subconjunctival hemorrhage were observed in patients with mature cataracts. Additionally, a single case of iris trauma was identified in a patient with NUC3.

To assess the impact of cataract severity (lens firmness), second-eye cataract surgery, and prolonged surgery duration on pain scores and patients’ cooperation, we conducted a post-hoc subgroup analysis, as presented in Table 3. Prolonged surgery (exceeding the 75th percentile of the entire population’s operative time) ranged from 22 to 40 min. The pain scores immediately after surgery were lower in patients in the Lidocaine group with NUC1–3 cataracts (*p* < 0.001) and those who underwent second-eye cataract surgery (*p* = 0.022). However, there was no statistically significant difference among patients with mature cataracts (*p* = 0.208) and those with prolonged procedure (*p* = 0.239). Despite this, the Lidocaine group exhibited a higher proportion of patients reporting no pain and a lower proportion experiencing moderate pain during prolonged phacoemulsification (28.6% with no pain and 7.1% with moderate pain) compared to the Control group (9.1% with no pain and 27.3% with moderate pain). The patient cooperation score was relatively comparable between the groups for patients with NUC1–3 cataracts (score difference [95% CI] = − 010: [− 0.34, 0.14], *p* = 0.402) and for patients who received second-eye cataract surgery (score difference [95% CI] = − 0.15 [− 0.60, 0.30], *p* = 0.495). Conversely, among patients with mature cataracts and prolonged operation, the patient cooperation score was significantly better in the Lidocaine group (Mature cataracts subgroup: score difference [95% CI] = − 0.82 [− 1.47, − 0.16], *p* = 0.016, Prolonged operation subgroup: score difference [95% CI] = − 1.14 [− 1.92, − 0.35], *p* = 0.006).

**Table 3:** Subgroup analysis of immediate postoperative pain and cooperation scores between the study groups

	Control group, n (%)	Lidocaine group, n (%)	*p* ^†^
	**Subgroup: Nuclear cataract 1–3**		
	***n*** **= 70**	***n*** **= 61**	
Immediate postoperative pain			< 0.001
- No pain	10 (14.3)	28 (45.9)	
- Mild	47 (67.1)	31 (50.8)	
- Moderate	11 (15.7)	2 (3.3)	
- Severe	2 (2.9)	0 (0)	
Cooperation score^*^ (Median [IQR])	0 (0, 1)	0 (0, 1)	0.402
- Score difference (95% confidence interval)		−0.10 (− 0.34, 0.14)	
	**Subgroup: Mature cataracts**		
	***n*** **= 18**	***n*** **= 27**	
Immediate postoperative pain			0.208
- No pain	4 (22.2)	10 (37.0)	
- Mild	13 (72.2)	17 (63.0)	
- Moderate	1 (5.6)	0 (0)	
- Severe	0 (0)	0 (0)	
Cooperation score^*^ (Median [IQR])	1 (0, 2)	0 (0, 1)	0.016
- Score difference (95% confidence interval)		−0.82 (− 1.47, − 0.16)	
	**Subgroup: Second-eye operation**		
	***n*** **= 26**	***n*** **= 26**	
Immediate postoperative pain			0.022
- No pain	4 (15.4)	10 (38.5)	
- Mild	17 (65.4)	16 (61.5)	
- Moderate	4 (15.4)	0 (0)	
- Severe	1 (3.8)	0 (0)	
Cooperation score^*^ (Median [IQR])	0 (0, 1)	0 (0, 1)	0.495
- Score difference (95% confidence interval)		−0.15 (− 0.60, 0.30)	
	**Subgroup: Prolonged operation** ^**‡**^		
	***n*** **= 11**	***n*** **= 14**	
Operative time (minutes) (Median [IQR])		25 (24, 28)	25 (23, 33)
Immediate postoperative pain			0.239
- No pain		1 (9.1)	4 (28.6)
- Mild		7 (63.6)	9 (64.3)
- Moderate		3 (27.3)	1 (7.1)
- Severe		0 (0)	0 (0)
Cooperation score^*^ (Median [IQR])	2 (1, 2)	0 (0, 1)	0.006^†^
- Score difference (95% confidence interval)		−1.14 (− 1.92, -0.35)	

^*^ The patient cooperation score ranged 0–5. A score of 5 indicated the worst cooperation, whereas a score of 0 denoted the best cooperation

^†^ The reported results were derived from ordinal logistic regression analysis for immediate postoperative pain and linear regression with robust standard errors for the cooperation score

^‡^ Prolonged operation = patients who had operative time exceeding the 75th percentile of the entire population’s operative time

IQR, interquartile range

Patients in both groups successfully underwent phacoemulsification procedures in this study, with only some patients requiring additional topical anesthesia.

## Discussion

Our study demonstrated the feasibility of using topical anesthesia techniques with or without subconjunctival lidocaine injection to perform phacoemulsification procedures in senile cataract treatment. Successful outcomes were achieved in all surgeries. An additional lidocaine injection reduced immediate postoperative pain (the percentages of patients with mild, moderate, and severe pain were 68.2%, 13.6%, and 2.3%, respectively, in the Control group, versus 54.5%, 2.3%, and 0%, respectively, in the Lidocaine group [*p* < 0.001]). The difference in pain scores also persisted after 2 h (the percentages of patients with no pain, mild pain, and moderate pain were 43.2%, 54.5%, and 2.3%, respectively, in the Control group, compared with 63.6%, 33.0%, and 3.4%, respectively, in the Lidocaine group [*p* = 0.011]). However, the lidocaine injection itself was significantly painful (*p* = 0.001). The acidic nature of lidocaine could have contributed to this discomfort, as patients in the Control group received a subconjunctival injection of balanced salt solution (neutral pH). Buffering lidocaine with sodium bicarbonate might not be feasible because of the small volume of 0.2 ml required for the injection [23]. Our study results were in line with prior findings, suggesting that topical anesthesia is associated with increased pain. However, these studies also demonstrated that all phacoemulsification procedures can be successfully completed using topical anesthesia alone without the need for additional methods, consistent with our findings [6, 12, 14]. Interestingly, a meta-analysis of 15 studies revealed that patients significantly preferred topical anesthesia over regional anesthesia [6].

Subconjunctival lidocaine injection was associated with a higher occurrence of anesthetic technique-related subconjunctival hemorrhage (Lidocaine vs. Control: 30.7% vs. 6.8%, *p* < 0.001). The incidence of subconjunctival hemorrhage in our study aligns with data reported in a previously published study (24.4%) [24]. Because patients in both groups received a subconjunctival injection, the increased incidence of subconjunctival hemorrhage in the Lidocaine group could be partially attributed to mechanical trauma from the injection. We hypothesized that the higher rate of subconjunctival hemorrhage in the Lidocaine group was attributable to blood vessel injury resulting from the drug or its acidic solution [25]. Subconjunctival hemorrhage is an acceptable complication because it is self-limited, does not require treatment, and does not result in long-term consequences [26]. Although scleral perforation associated with subconjunctival injection has been reported, this complication is extremely rare, and it did not occur in our study [27].

The addition of subconjunctival lidocaine injection both reduced postoperative pain and significantly improved patients’ cooperativity (score difference [95% CI]: −0.25 [− 0.49, − 0.01]). Our findings are consistent with those of previous studies highlighting the correlation between better pain control and improved patient cooperation [28, 29]. Patient cooperation could be especially crucial for ophthalmologists during their learning curve periods [30]. Moreover, in developing countries, in which the lack of anesthesiologists [31] requires ophthalmologists to perform both anesthesia and surgery, patient cooperation assumes even greater importance. Lower patient cooperation was also associated with a higher frequency of complications [32]. Although topical anesthesia is a simple technique [33], surgeons must carefully weigh the trade-off between cooperation and reduced postoperative pain against the simplicity of the procedure and potential patient discomfort during subconjunctival injection.

Three potential factors could influence anesthetic properties: the impact of hard cataracts [20], whether the ongoing surgery was the second-eye cataract surgery [21], and prolonged phacoemulsification [22]. We conducted subgroup analyses to investigate these factors. Concerning the influence of hard cataracts, the additional lidocaine injection offered less immediate postoperative pain, albeit without statistical significance among patients with mature cataracts (*p* = 0.208). One interpretation is that the consistency of cataracts lowers the efficacy of the subconjunctival injection, whereas previous research suggested that patients with better visual acuity experience an increased level of pain perception [28]. However, our subgroup analysis also revealed that adding lidocaine to topical anesthesia in the mature cataract subgroup led to better patient cooperation (score difference [95% CI]: −0.82 [− 1.47, − 0.16], *p* = 0.016). Considering this, the addition of lidocaine could yield benefits in patients with mature cataracts, particularly in terms of patient cooperation. This is especially relevant because phacoemulsification is more challenging in patients with more severe cataracts [34]. Patients undergoing second-eye surgery reported more pain and less cooperation [21]. Our subgroup analysis revealed that the addition of lidocaine significantly reduced immediate postoperative pain (lower percentage of moderate and severe pain, *p* = 0.022) without affecting the cooperation score. Additionally, we analyzed the effect of prolonged operative time. The Lidocaine group showed a trend toward a more prolonged anesthetic effect, demonstrating better immediate postoperative pain proportions (lower moderate pain and higher no pain, *p* = 0.239), along with a significantly improved cooperation score (*p* = 0.006). These findings suggest that adding subconjunctival anesthesia injection to topical anesthesia alone might offer more benefits for challenging cases, such as higher-grade cataracts, second-eye operations, or prolonged procedures, especially in terms of reducing pain and improving patients’ cooperation. However, we cannot draw definitive conclusions from the subgroup analysis results due to the limited sample size.

This study possesses a significant strength in its double-blinded randomized trial design, enabling an effective evaluation of the efficacy of adding subconjunctival lidocaine to topical anesthesia for phacoemulsification in patients with senile cataracts. The results provide valuable insights for ophthalmologists choosing between topical anesthesia alone and in combination with subconjunctival lidocaine injection. Additionally, our results might be particularly advantageous for developing countries in which the availability of anesthesiologists is limited.

This study was limited by its reliance on outcomes from a single surgeon (WW) [35]. However, the surgeon had more than 5 years of experience in phacoemulsification, and regularly performed both anesthetic techniques. Therefore, we believe that the study findings can be extrapolated to ophthalmologists experienced in performing phacoemulsification procedures. Another limitation of the study stems from its design as a clinical trial, thereby limiting the generalizability of findings to a broader patient spectrum. More complicated cases, which might involve different expected treatments affecting postoperative pain, were excluded according to the exclusion criteria. Caution is warranted in extending the study results to patients closely matching our exclusion criteria, particularly those exhibiting poor cooperation during preoperative examination, difficult communication, a propensity for or presence of lens subluxation, or advanced (brunescent) cataracts. Finally, since the sample size for this study was calculated based on the primary outcome, which was immediate postoperative pain, the results of the secondary outcomes should be interpreted with caution, considering both the degree of significance and the size of the differences. We chose not to conduct a post-hoc power analysis for the secondary outcomes, as it could be highly invalid and misleading [36].

## Conclusions

The addition of subconjunctival lidocaine to topical anesthesia reduced postoperative pain and improved cooperation in patients with senile cataracts undergoing phacoemulsification. However, the subconjunctival lidocaine injection was associated with more pain during the injection and an increased occurrence of subconjunctival hemorrhage. Careful consideration is necessary to weigh the trade-off between risks and benefits. Both topical anesthesia alone and the addition of subconjunctival lidocaine can successfully facilitate phacoemulsification procedures.

## Data Availability

The datasets used and/or analysed during the current study are available from the corresponding author on reasonable request.

## Abbreviations

CI: confidence interval
NRS: numeric rating scale
NUC: nuclear cataract
